# COL6A6 interacted with P4HA3 to suppress the growth and metastasis of pituitary adenoma via blocking PI3K-Akt pathway

**DOI:** 10.18632/aging.102300

**Published:** 2019-10-17

**Authors:** Ruiqing Long, Zhuohui Liu, Jinghui Li, Hualin Yu

**Affiliations:** 1 Department of Neurosurgery, The First Affiliated Hospital of Kunming Medical University, Kunming 650032, Yunnan, China; 2 Department of Otolaryngology, The First Affiliated Hospital of Kunming Medical University, Kunming 650032, Yunnan, China

**Keywords:** pituitary adenoma, COL6A6, P4HA3, PI3K-Akt pathway, invasion

## Abstract

The role and mechanism of collagen type VI alpha 6 (COL6A6) on tumor growth and metastasis in pituitary adenoma (PA) was determined. COL6A6 was downregulated in PA tissues and cell lines, which was negatively associated with the expression of prolyl-4-hydroxylase alpha polypeptide III (P4HA3) in the progression of PA. Overexpression of COL6A6 significantly suppressed tumor growth and metastasis capacity in PA. In addition, P4HA3 worked as the upstream of the PI3K-Akt pathway to alleviate the antitumor activity of COL6A6 on the growth and metastasis of both AtT-20 and HP75 cells. Furthermore, the inhibitory effect of COL6A6 on cell proliferation, migration and invasion, and epithelial-mesenchymal transition (EMT) was reversed by P4HA3 overexpression or activation of the PI3K-Akt pathway induced by IGF-1 addition, which provided a new biomarker for clinical PA treatment.

## INTRODUCTION

Pituitary adenoma (PA) is a kind of common disease in clinical endocrinology and neurology, whose incidence is second only to gliomas and meningiomas and listed in the third place among the intracranial tumors [1, 2]. Meanwhile, accounting for about 45-55% of PA often invade cavernous sinus, sphenoid sinus, internal carotid artery and other important tissue structure, which leads to a failure of complete removal by surgical resection and high incidence of recurrence, known as invasive PA [3, 4]. In addition, few reliable biomarkers to predict the development and progression of PA. Therefore, it is important to find a new biomarker for improving its clinical treatment and early diagnosis.

At present, numerous studies have shown that tumor suppressor genes serve an important role in regulating tumor etiology, development, and prognosis [5–8]. It is expected to seek the targeted gene among the tumor suppressor genes for gene therapy. Discovering and elucidating the action mechanism of related genes is not only helpful in finding markers that can reflect the specific tumor biological for clinical diagnosis and follow-up study, but also can provide theory basis for developing the new therapy of this kind of tumor [9, 10]. Collagen type VI (COL6) is mainly composed of several different alpha chains, which serves an important role in regulating cell proliferation, apoptosis, invasion, and metastasis, epithelial-mesenchymal transition (EMT) and angiogenesis of malignant tumors by the formation of cell microfilament network and interaction with extracellular matrix molecules [11, 12]. For example, Willumsen et al. reported that COL6 fragments were upregulated in the plasma of patients with cancer [13]. Overexpression of COL6A3 promoted the process of EMT in bladder cancer [14]. Moreover, the present study showed that COL6A6 was upregulated in PA tissues from microarray GSE26966. However, the role and mechanism of COL6A6 in cancer progression has not yet clear.

In the present study, the expression levels of COL6A6 in PA tissues and cell lines were examined, which may regulate the malignant biological behavior of PA cells by interacting with P4HA3 and further regulating the PI3K-Akt pathway *in vitro*. Furthermore, the effects of COL6A6 interacted with P4HA3 on tumor growth and metastasis in PA were substantiated *in vivo*. Taken together, the aim of this study was to determine the role and mechanisms of COL6A6 in PA progression.

## RESULTS

### The differently expressed genes and signaling pathway in PA

**Figure 1:**
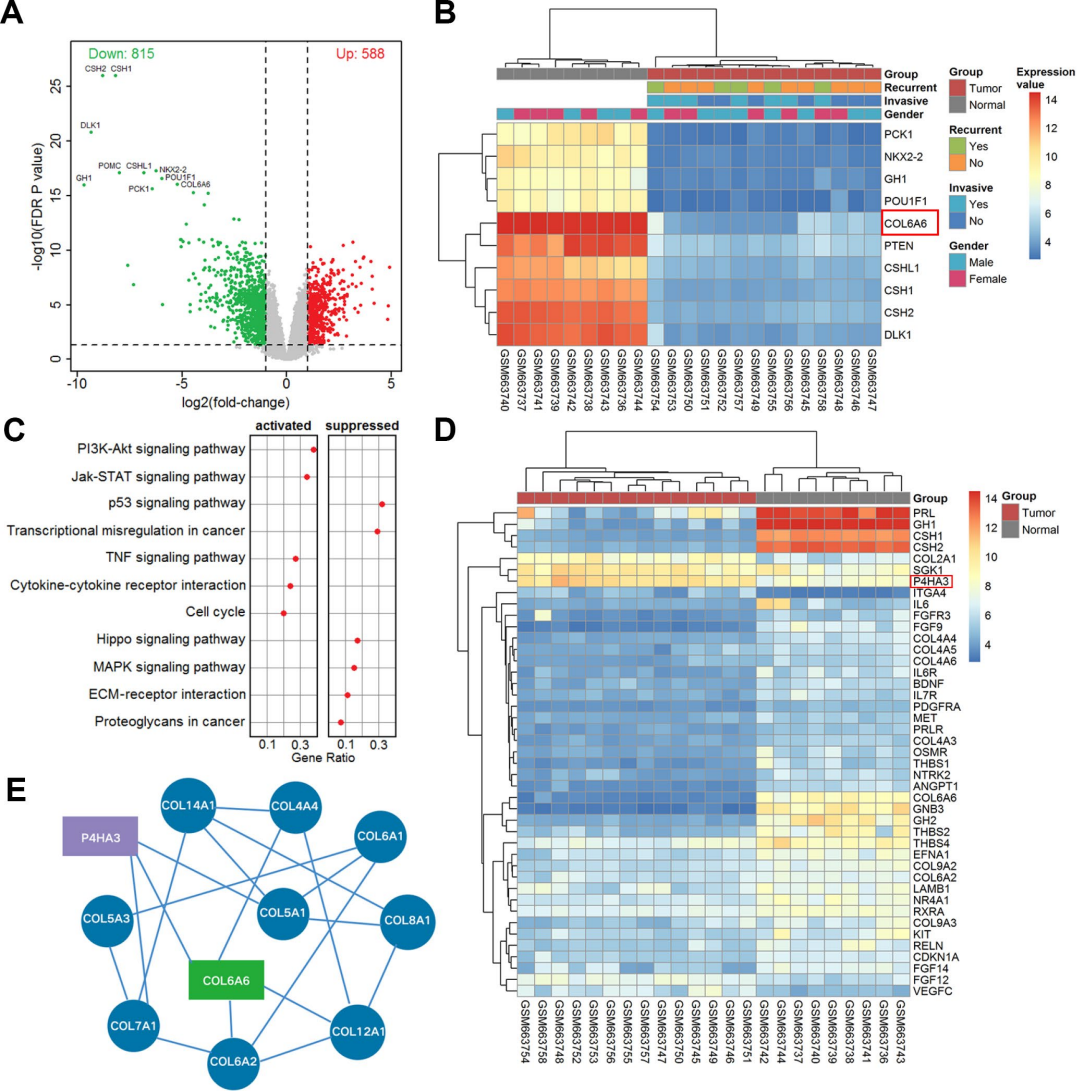
**The differentially expressed mRNAs and pathway in PA.** (**A**) Volcano plot of differentially expressed genes in the whole cohort vs. normal. The red dots represent up-regulated genes and the green dots represent down-regulated genes; (**B**) Heatmap of top ten differentially expressed genes in tumor vs. normal; (**C**) The dotplot of significantly altered pathways in PA tissues analyzed by GSEA; (**D**) The heat map of differentially expressed mRNAs in the PI3K-Akt pathway analyzed from microarray GSE26966; (**E**) The correlative network of COL6A6 and differentially expressed mRNAs involved in the PI3K-Akt signaling pathway in PA.

According to the above criteria, we finally chose 9 normal pituitary and 14 PA samples (GSE26966) for our re-analysis by Affymetrix Human Genome U133 Plus 2.0 Array. The results showed that the top 10 significantly downregulated genes (CSH1, CSH2, DLK1, NKX2-2, CSHL1, PTEN, POU1F1, COL6A6, GH1, and PCK1) associated with tumor metastasis were labeled in Figure 1A, 1B. Meanwhile, the expression of COL6A6 in PA tissues was the lowest (Figure 1B). Besides, the dotplot of GSEA showed that PI3K-Akt signaling pathway was notably activated in all comparisons (Figure 1C), and most of the genes associated with the PI3K-Akt pathway were downregulated (Figure 1D). Of note, P4HA3 acts as an upstream regulator gene of the PI3K-Akt pathway, was upregulated in PA tissues compared with the normal group (Figure 1D). Furthermore, COL6A6 was found to be closely related to P4HA3 in the networks of differentially expressed genes (Figure 1E).

### Expression of COL6A6 and P4HA3 in PA and its correlation with tumorigenesis

**Figure 2:**
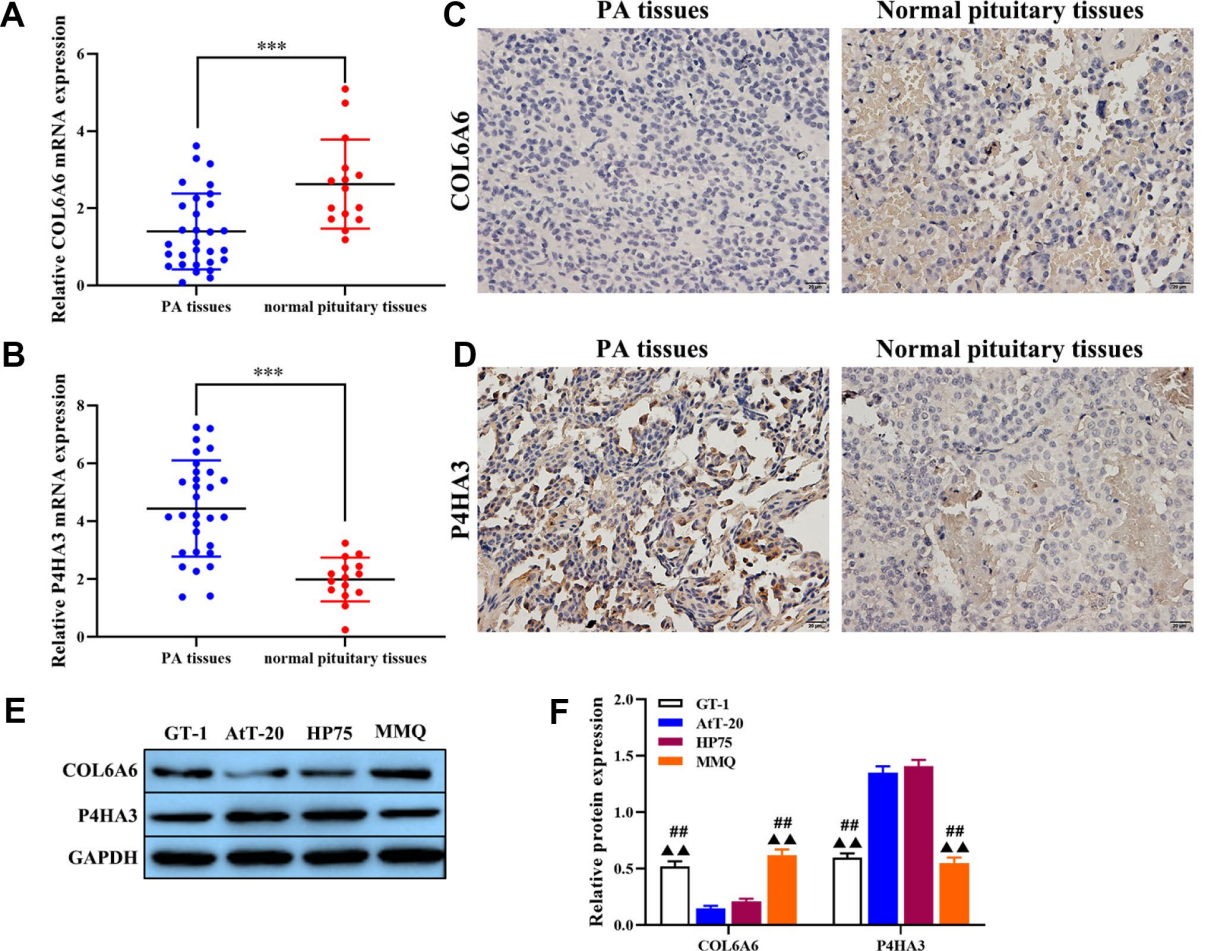
**The expression of COL6A6 and P4HA3 in PA tissues and cell lines.** (**A** and **B**) RT-qPCR was used to detect the expression of COL6A6 and P4HA3 in PA tissues and normal pituitary tissues; (**C** and **D**) Immunohistochemistry staining was applied to detect the expression of COL6A6 and P4HA3 in PA tissues and normal pituitary tissues; (**E** and **F**) Western blotting was used to evaluate the expression of COL6A6 and P4HA3 in PA cell lines. ^***^P<0.001, compared with normal pituitary tissues; ^##^P<0.01, compared with AtT-20 cells; ^▲▲^P<0.01, compared with HP75 cells.

Based on the regulated network of COL6A6 and P4HA3 in PA, the expression of COL6A6 and P4HA3 in PA and its correlation with tumorigenesis was investigated. As shown in Table 1, COL6A6 was a negative correlation with tumor size and high grade. RT-qPCR analysis results showed that COL6A6 was downregulated in PA tissues compared with the normal pituitary tissues (all P<0.001, Figure 2A), and P4HA3 was upregulated in PA tissues (P<0.001, Figure 2B), which consistent with the results of immunohistochemistry staining (Figure 2C, 2D). Furthermore, COL6A6 were markedly decreased, and P4HA3 upregulated in PA cell lines by western blotting, especially in AtT-20 and HP75 cells (all P<0.01, Figure 2E, 2F). Thus, low-expression of COL6A6 and P4HA3 overexpression may be associated with the progression of PA. Meanwhile, AtT-20 and HP75 cells were chosen for subsequent experiments.

**Table 1:** The correlation between clinical features of PA and the expression of COL6A6.

**Clinical features**	**Number**	**COL6A6 expression**		**P value**
	30	High 13(43.33%)	^a^Low 17(56.67%)	
**Age**				0.464
≥50	16(53.33%)	7(23.33%)	9(30.00%)	
<50	14(46.67%)	6(20.00%)	8(26.67%)	
**Sex**				0.491
Male	16(53.33%)	6(20.00%)	10(23.33%)	
Female	14(46.67%)	7(23.33%)	7(23.33%)	
**Tumor size**				0.035
≥3 cm	18(60.00%)	5(16.67%)	13(43.33%)	
<3 cm	12(40.00%)	8(26.67%)	4(13.33%)	
**Knosp classification**				0.013
Grad 0~2	11(36.67%)	8(26.67%)	3(10.00%)	
Grad 3~4	19(63.33%)	5(16.67%)	14(46.66%)	
**Type of adenomas**				0.984
^b^PRL	14(46.67%)	6(20.00%)	8(26.67%)	
^c^CH	6(20.00%)	3(10.00%)	3(10.00%)	
^d^ACTH	5(16.67%)	2(6.64%)	3(10.00%)	
^e^ NFPA	5(16.67%)	2(6.64%)	3(10.00%)	

^a^The median of relative COL6A6 expression level is 1.40, so the number of low COL6A6 expression is 17 (<1.40).^b^PRL: Prolactinoma; ^c^CH: Growth hormone pituitary adenoma; ^d^ACTH: Corticotroph pituitary adenoma; ^e^NFPA: Nonfunctioning pituitary adenoma.

### Effect of COL6A6 on the growth and metastasis of PA cells *in vitro* and *in vivo*

**Figure 3:**
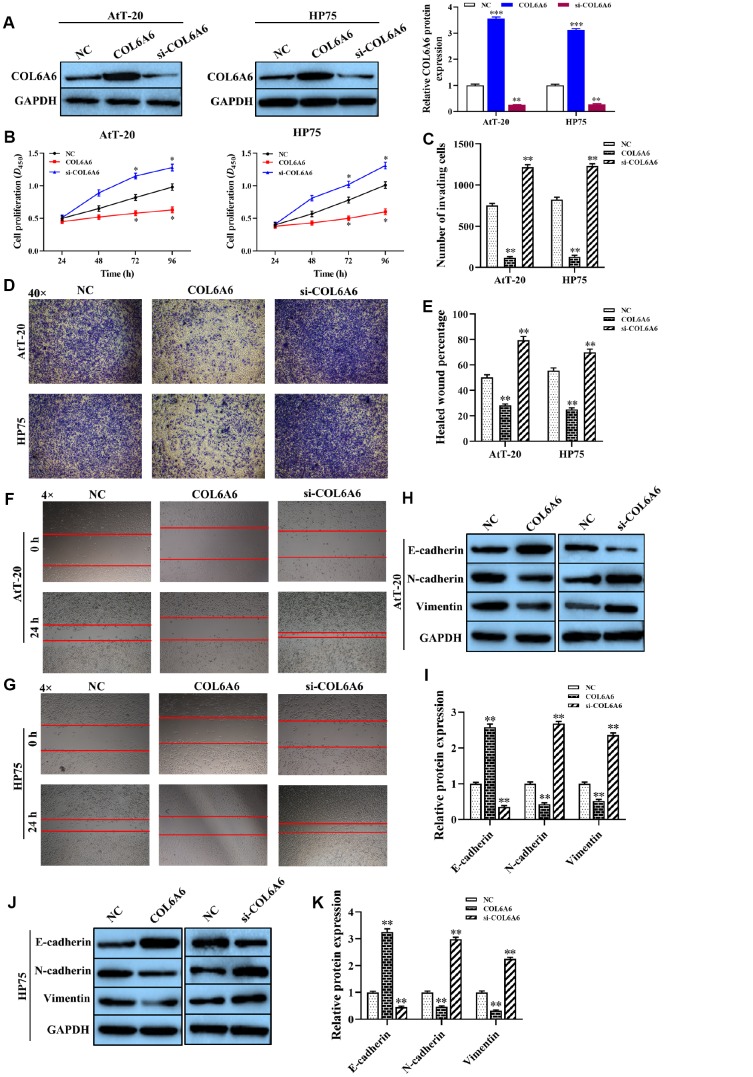
**The effect of COL6A6 on the proliferation, invasion, migration, and EMT of AtT-20 and HP75 cells**. (**A**) The expression of COL6A6 protein was determined by western blotting; (**B**) The proliferation ability of AtT-20 and HP75 cells were measured by CCK-8 assay; (**C** and **D**) The invasion of AtT-20 and HP75 cells were measured by Transwell assay (40×); (**E**–**G**) The migration ability of AtT-20 and HP75 cells were evaluated by wound healing assay (4×); (**H**–**K**) The expression of EMT related proteins in AtT-20 and HP75 cells were detected by western blotting. ^*^P<0.05, ^**^P<0.01, ^***^P<0.001, compared with the NC group.

To explore the effect of COL6A6 on the proliferation, invasion, migration, and apoptosis of AtT-20 and HP75 cells. Western blotting analysis results showed that the expression level of COL6A6 protein was increased or decreased in AtT-20 and HP75 cells when transferred with pcDNA-COL6A6 (both P<0.001) or si-COL6A6 (both P<0.01) compared with the NC group (Figure 3A). CCK-8 analysis results showed that overexpression of COL6A6 notably inhibited the proliferation ability of both AtT-20 and HP75 cells compared with the NC group (both P<0.05, Figure 3B). Besides, the effect of COL6A6 knockdown on the proliferation of both AtT-20 and HP75 cells were detected (both P<0.05, Figure 3B). Transwell and wound healing assay demonstrated that upregulation of COL6A6 remarkably inhibited invasion and migration capacity of both AtT-20 and HP75 cells (all P<0.01, Figure 3C–3G). Conversely, knockdown of COL6A6 promoted the invasion and migration of both At T-20 and HP75 cells (all P<0.01, Figure 3C–3G). Western blotting showed that COL6A6 elevated significantly suppressed the expression levels of N-cadherin and Vimentin proteins in AtT-20 and HP75 cells compared with NC group (all P<0.01, Figure 3H–3K), but enhanced the E-cadherin protein (all P<0.01, Figure 3H–3K). However, silencing of COL6A6 got opposite results (all P<0.01, Figure 3H–3K). These results strongly implied that overexpression of COL6A6 was suppressed the proliferation, invasion, migration, and EMT of AtT-20 and HP75 cells.

**Figure 4:**
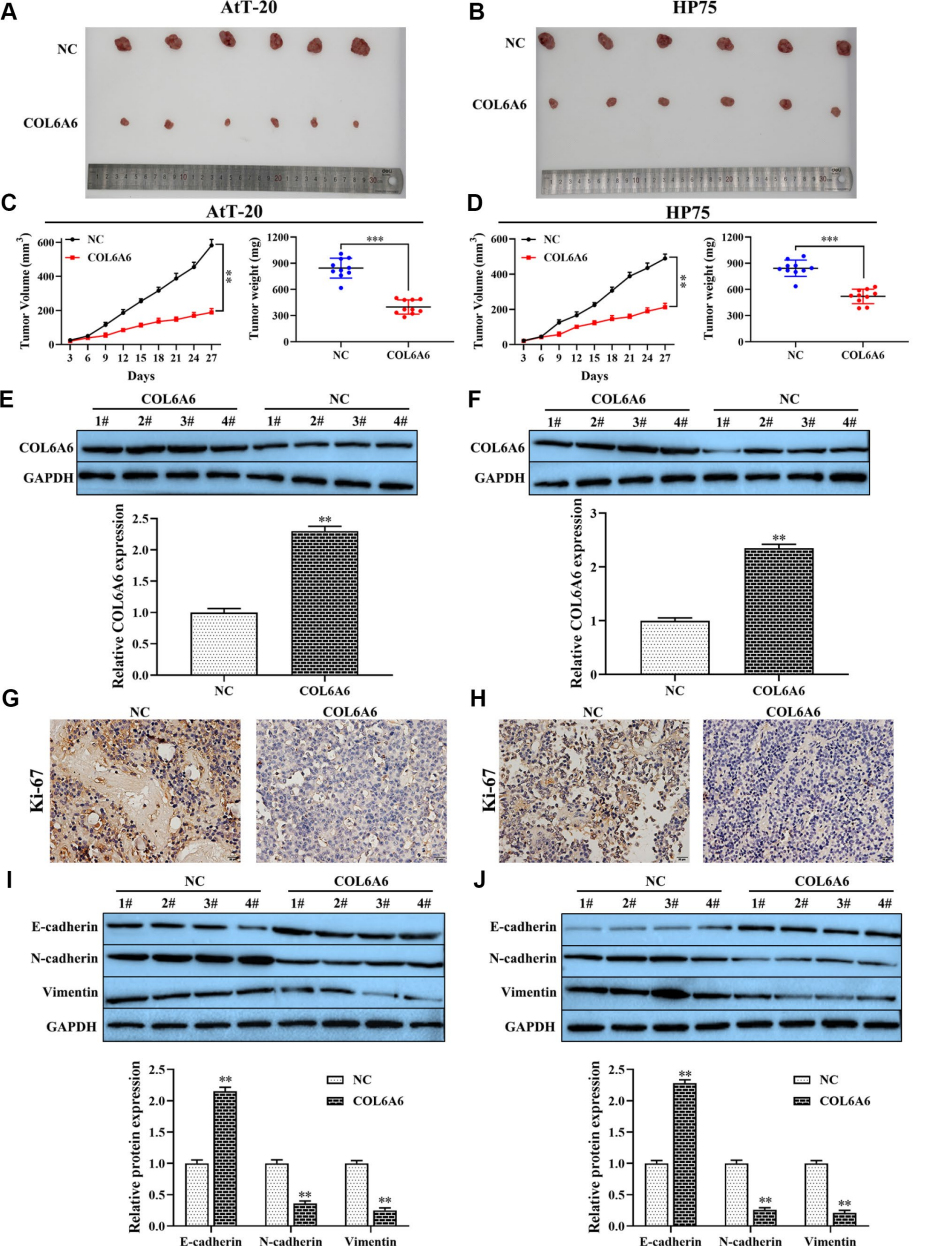
**The effect of COL6A6 on the growth and metastasis of xenograft tumor**. (**A** and **B**) The tumor size was obtained from nude mice; (**C** and **D**) Tumor volume and tumor weight of nude mice were analyzed; (**E** and **F**) The expression of COL6A6 in xenograft tumor tissues were detected by western blotting; (**G** and **H**) The expression of Ki-67 in xenograft tumor tissues were detected by immunohistochemistry staining; (**I** and **J**) The expression of E-cadherin, vimentin, and N-cadherin were measured by western blotting. ^**^P<0.01, ^***^P<0.001, compared with NC group.

To further examine the effect of COL6A6 on the growth and metastasis of PA *in vivo*. Overexpression of COL6A6 noticeably inhibited the size of the tumor, tumor volume (all P<0.01) and tumor weight (all P<0.001) compared with the NC group (Figure 4A–4D). Moreover, western blotting analysis results showed that COL6A6 elevated markedly increased COL6A6 protein level in tumor tissues of PA mice model compared with the NC group (all P<0.01, Figure 4E, 4F). Immunohistochemistry results demonstrated that upregulation of COL6A6 decreased the expression of Ki-67 in xenograft tumor tissues (Figure 4G, 4H). Furthermore, compared with the NC group, overexpression of COL6A6 significantly suppressed the expression levels of N-cadherin and Vimentin protein, while the expression level of E-cadherin was upregulated (all P<0.01, Figure 4I, 4J). Therefore, our finding suggest that upregulation of COL6A6 may restrict PA growth and metastasis *in vitro* and *in vivo*.

### Effect of COL6A6 on the PI3K-Akt signaling pathway through interacting with P4HA3

**Figure 5:**
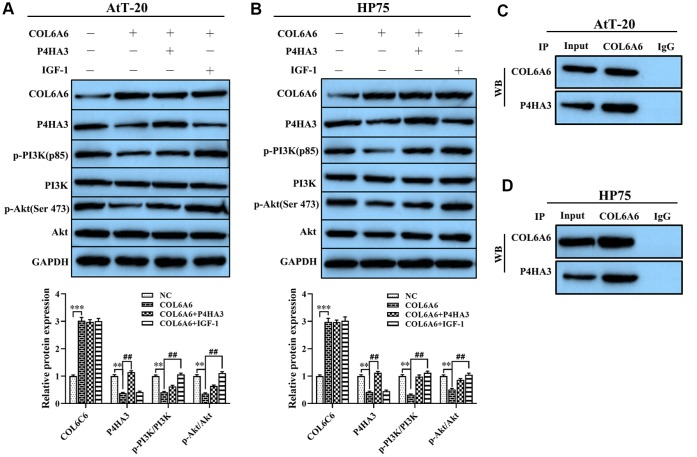
**Effect of COL6A6 on the PI3K-Akt signaling pathway through interacting with P4HA3**. (**A** and **B**) Western blotting was used to detect the expression of COL6A6, P4HA3, PI3K-Akt pathway-related protein in AtT-20 and HP75 cells; (**C** and **D**) Coimmunoprecipitation to validate the COL6A6-P4HA3 interaction. ^**^P<0.01, ^***^P<0.001, compared with NC group; ^##^P<0.01, compared with COL6A6 group.

To further clarify the mechanism of COL6A6 regulated the growth and metastasis of PA cells, we assessed the effect of COL6A6 interacted with P4HA3 on the PI3K-Akt pathway, which plays an important role in regulating tumor development, tumor cells invasion and EMT. Western blotting showed that COL6A6-elevated significantly decreased the expression of P4HA3 protein, as well as decreased the expression of p-PI3K and p-Akt, but the expression of PI3K and Akt remained unchanged (all P<0.01, Figure 5A, 5B). In addition, we further detected the expression p-PI3K and p-Akt in both AtT-20 and HP75 cells treated with the PI3K-Akt pathway activator (IGF-1) or P4HA3 overexpression, the results showed that P4HA3 upregulation or IGF-1 treatment abolished the inhibitory effect of COL6A6-elevated on the PI3K-Akt pathway (all P<0.01, Figure 5A, 5B). Furthermore, co-immunoprecipitation showed that COL6A6 was closely related to P4HA3 (Figure 5C, 5D). These data indicated that COL6A6 may serve an anti-oncogene role in the development of PA by interacting with P4HA3 and further blocking the PI3K-Akt pathway, while its mechanism needs further verification.

### Effect of COL6A6 regulated growth and metastasis of PA cells through mediating P4HA3-PI3K-Akt pathway

**Figure 6:**
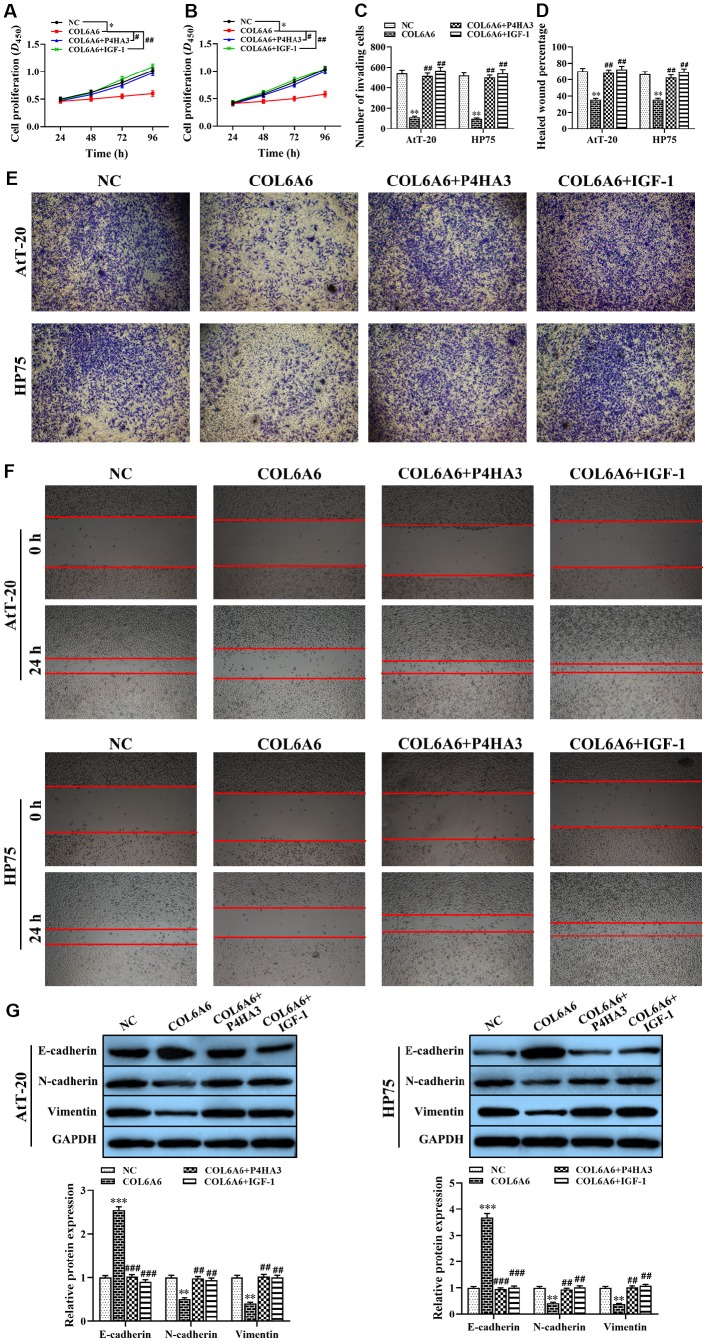
**The effect of COL6A6 interacted with P4HA3 on the proliferation, invasion, migration, and EMT of AtT-20 and HP75 cells through regulating the PI3K-Akt pathway.** (**A** and **B**: The proliferation ability of AtT-20 and HP75 cells were measured by CCK-8 assay; (**C** and **E**) The invasion of AtT-20 and HP75 cells were measured by Transwell assay (40×); (**D** and **F**) The migration ability of AtT-20 and HP75 cells were evaluated by wound healing assay (4×); (**G**) The expression of EMT related proteins in AtT-20 and HP75 cells were detected by western blotting. ^*^P<0.05, ^**^P<0.01, ^***^P<0.001, compared with NC group; ^#^P<0.05, ^##^P<0.01, ^###^P<0.001, compared with COL6A6 group.

**Figure 7:**
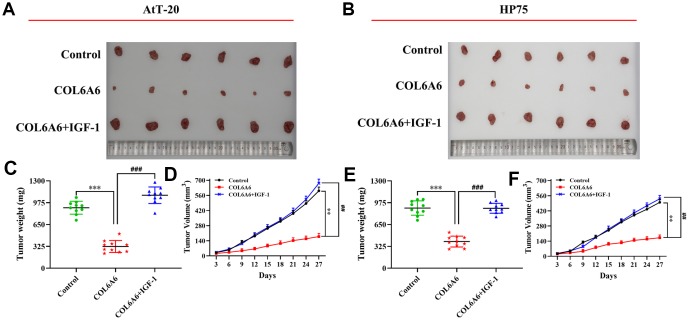
**The effect of COL6A6 on the growth and metastasis of xenograft tumor by mediating PI3K-Akt pathway.** (**A** and **B**) The tumor size was obtained from nude mice; C and E: The tumor weight was measured; (**D** and **F**) The tumor volume curve of nude mice was analyzed. ^**^P<0.01, ^***^P<0.001, compared with control group; ^##^P<0.01, ^###^P<0.001, compared with COL6A6 group.

Based on the effect of COL6A6 on the PI3K-Akt signaling pathway by interacting with P4HA3 *in vitro*, to further explore the role of COL6A6 and P4HA3 mediated the proliferation, invasion, migration, and apoptosis of both AtT-20 and HP75 cells by regulating PI3K-Akt pathway. CCK-8, Transwell and wound healing assay demonstrated that P4HA3 overexpression or IGF-1 treatment significantly abolished upregulation of COL6A6 exerted antitumor activity in both AtT-20 and HP75 cells (P_proliferation_<0.05, P_invasion_<0.01, P_migration_<0.01, Figure 6A–6F). Besides, we found that IGF-1 addition or P4HA3-elevated reversed the inhibitory effect of COL6A6 on the EMT of both AtT-20 and HP75 cells (all P<0.001, Figure 6G). Furthermore, we observed that overexpression of COL6A6 significantly decreased the size of the tumor compared with the control group (all P<0.001, Figure 7A, 7B), while the size of the tumor with IGF-1 treatment was bigger than the COL6A6 overexpression group (all P<0.001). Meanwhile, IGF-1 rescued the antitumor activity of COL6A6 in subcutaneous xenograft models (Figure 7C–7F). Taken together, these data suggested COL6A6 elevated inhibited tumor growth and metastasis in PA via interacting with P4HA3 and blocking the PI3K-Akt pathway.

## DISCUSSION

PA is a benign tumor, which accounts for about 10% of intracranial tumors [15]. Because of the special biological morphology and behavior of PA, it is difficult to completely remove the tumor, which leads to a high recurrence rate of the tumor after surgery [16]. Therefore, the identification of biomarker to regulate the development and progression of PA has become an urgent issue in clinical treatment. In this study, we found that the expression COL6A6 was downregulated in PA tissues and cell lines, which may be a biomarker for PA prognosis. Moreover, overexpression of COL6A6 significantly inhibited cell proliferation, invasion, migration and induced apoptosis of PA cells by blocking the PI3K-Akt pathway. Furthermore, COL6A6-elevated suppressed tumor growth and metastasis in PA.

COL6 is beaded fibrous collagen located near the basement membrane, which is involved in maintaining the transformation of epithelial cells and endothelial cells. According to previous studies, COL6 fragments were aberrantly expressed in multiple solid tumors [17]. For example, the expression of COL6A3 and COL6A1 was upregulated in most cancer types compared with controls [13], which play an important role in regulating the tumorigenesis and metastasis of cancer. Burchardt et al. confirmed that COL6A1 and COL6A2 were upregulated in serum from patients with melanoma [18]. Kang et al. showed that COL6A3 was significantly upregulated in pancreatic ductal adenocarcinoma [19]. Another study found that COL6A3 was a potential prognosis marker of colorectal cancer, which was upregulated in cancer tissues [20]. These data suggested that COL6 exerted a biomarker potential of the progression of tumors. Moreover, COL6 fragments have been reported to be involved in other types of disease, including lung function changes [21], muscular dystrophy [22, 23] and Bethlem myopathy [24], etc. To our knowledge, there are few studies confirmed the role of COL6 in the progression of multiple tumors. For instance, Yeh et al. reported that high COL6A6 expression level were significantly correlated with early pathological stage in breast cancer [25], but the biological function of COL6A6 in the progression of multiple tumors remains unclear. Interestingly, our data showed that COL6A6 was lowly expressed in the PA tissues compared with healthy samples by microarray analysis. At the same time, we verified the gain- or loss-of-function of COL6A6 in the biological behavior of PA cells.

Recently, several studies found that P4HA3, as an oncogene in multiple tumors, was a positive correlation with tumor growth and poor survival [26]. Another study based on the bioinformatic analysis in The Cancer Genome Atlas (TCGA) found that P4HA3 upregulation is highly correlated with genes representing ECM production in breast cancer, and higher P4HA3 expression is associated with a worse prognosis [27]. Meanwhile, Song et al. found that P4HA3 was significantly overexpressed in gastric cancer tissues compared with adjacent tissues, and which was associated with gastric cancer cell metastasis [28]. However, the role and mechanism of P4HA3 in the malignant tumors was largely unknown. As expected, our finding showed that P4HA3 was upregulated in PA tissues and cell lines, and overexpression of P4HA3 significantly alleviated the inhibitory effect of COL6A6 on the cell proliferation, migration, invasion and EMT in both PA cells. Mechanically, COL6A6 interacted with P4HA3 to suppress the growth and metastasis of pituitary adenoma via blocking PI3K-Akt pathway.

Over the past decades, PI3K-Akt signaling pathway has been proved that it plays an important regulatory role in the process of cell survival and growth, metabolic control, mitogenic signaling, cytoskeletal rearrangement, migration, and differentiation [29–32]. For example, inhibition of PI3K-Akt pathway suppressed the proliferation and metastasis of multiple tumor cells, including breast cancer [33], prostate cancer [34], endometrial cancer [35], non-small cell lung cancer [36], colorectal cancer [37] and PA [38]. Moreover, several studies confirmed that tumor suppressor or oncogene as a key player involved in the development and progression of malignant tumors through regulating PT3K-Akt pathway. Zhou et al. demonstrated that overexpression of miR-145 decreased the proliferation and invasion of PA cells by blocking the PI3K-Akt pathway [39]. Another study found that miR-106b promoted the proliferation and invasion of PA cells by targeting PTEN to activation of the PI3K-Akt pathway [38]. For the first time, we demonstrated that upregulation of COL6A6 suppressed growth and metastasis of PA cells by blocking the PI3K-Akt pathway *in vitro* and *in vivo*. Furthermore, PI3K is a key node regulating the PI3K-Akt pathway in cells. It has been proved that PI3Ks were potential targets for tumor therapy, especially PI3Kα as one of the most important targets of anti-tumors. For example, the addition of PI3Kα inhibitor BYL719 inhibited cell proliferation and induced apoptosis of triple-negative breast cancer [40]. Kim et al. showed that the PI3K inhibitor promoted cell death in Epstein-Barr virus-infected gastric cancer [41]. The above results suggested that suppression of PI3K-Akt pathway may be an effective therapeutic tool for treatment of PA patients.

## CONCLUSIONS

In summary, our data indicated that COL6A6 was downregulated in PA tissues and cell lines, which were well correlated with poor survival. COL6A6 inhibits PA cells growth and invasion through by inhibiting the PI3K-Akt pathway *in vitro* and *in vivo*. Furthermore, this study provided a new biomarker for PA clinical diagnosis and treatment.

## MATERIALS AND METHODS

### PA data collection

Microarray datasets of PA were searched and downloaded from the gene expression omnibus (GEO) database (http://www.ncbi.nlm.nih.gov/geo/). We conducted a rigorous screening for these datasets and the following are inclusion criteria: (1) the human microarray datasets were genome-wide; (2) samples in each study should include cases and controls; (3) tumor samples are non-cell line samples; and (4) raw data or expression matrix were available. According to the above criteria, we finally chose GSE26966 (Contributed by Michaelis KA et al. [42]) for our re-analysis. The dataset contains 9 normal pituitary and 14 PA samples that were tested by Affymetrix Human Genome U133 Plus 2.0 Array. In the PA samples, there were 7 invasive samples, 7 non-invasive samples, 5 recurrent samples, and 9 non-recurrent samples.

### Data pre-processing

R statistical software v3.4.1 was used to perform pre-processing and subsequent data analysis. We used the Robust Multichip Average (RMA) algorithm in the oligo package [43] to normalize the raw expression data and generate normalized gene expression intensity. We downloaded annotation platform GPL570 in GEO database for gene annotation and the methods see the previous publications [17, 18]. Finally, we got an expression matrix including 20307 genes and 23 samples.

### Differential expression gene analysis

Differentially expressed gene analysis was performed using the empirical Bayes algorithm in the “limma” package [46] in R. Differences (up- or down-regulated) were considered statistically significant for the absolute value of log2(fold-changes) higher than 1 and false discovery rate (FDR) adjusted P values ≤ 0.05. Differentially expressed genes were calculated between tumor and normal samples in five comparisons (whole cohort, invasive, non-invasive, recurrent, and non-recurrent). The expression profiles of differentially expressed genes were showed by heatmap in “pheatmap” package. We chose “ward. D2” algorithm to perform hierarchical clustering for samples in different groups. Venn diagram was used to compare the differentially expressed genes between these groups.

### Gene set enrichment analysis

We used javaGSEA desktop application v3.0 (http://software.broadinstitute.org/gsea/) [47] to perform gene set enrichment analysis (GSEA) of the five comparisons. Gene sets with less than 10 genes or more than 500 genes were excluded. The t-statistic mean of the genes was computed in each pathway using a permutation test with 1000 replications. Pathways with normalized enrichment scores (NESs) > 0 were considered up-regulated, and subsystems with NESs < 0 were considered down-regulated. Statistical significance was identified as FDR adjusted P values ≤ 0.05. We used “pathview” package [28] to display the enriched pathways and deregulated genes.

### Protein-protein interaction networks

STRING web server (https://string-db.org/cgi/input.pl) [49] was used to construct protein-protein interaction (PPI) networks of screened SAA effect genes. The parameters were set as follows: (1) the network edges were set as the molecular action (line shape indicates the predicted mode of action); (2) all types of active interaction sources were chosen, including text mining, experiments, databases, co-expression, neighborhood, gene fusion, and co-occurrence; (3) the minimum required interaction score was set at the medium confidence of 0.4; and (4) the maximum number of interactors in the first shell was the total number of query proteins only and the maximum number of interactors in the second shell was set to none. The enriched KEGG pathways of the input genes were automatically exported from the webserver.

### Tissue specimen

For the clinical aspect of the present study, 30 PA tissues and 15 normal pituitary tissues were collected. Tumor invasion was determined by Knosp garding scale. The present study complied with the principles of the Declaration of Helsinki and was approved by the regulations of the Ethics Committee of Kunming Medical University, as well as informed consent was obtained from patients. Samples were frozen in liquid nitrogen immediately and stored at -80°C for subsequent experiments.

### Cell culture and transfection

PA cell lines (HP75, AtT-20, GT-1, and MMQ) were purchased from the Shanghai Institutes for Biological Sciences of the Chinese Academy of Sciences and cultured according to the manufacturer’s protocols. Media was supplemented with 1% penicillin-streptomycin and 10% FBS (Gibco, USA) at 37°C and 5% CO_2_.

A total of 24 h prior to transfection, HP75 and AtT-20 cells were seeded in six-well plates at the optimum density and incubated overnight. pcDNA-COL6A6, sh-COL6A6, and control (blank plasmid) were transfected into both HP75 and AtT-20 cells using Lipofectamine^®^ 3000 reagent and Opti-MEM medium (Invitrogen Life Technologies, USA) according to the manufacturer’s protocol. The pcDNA-COL6A6, sh-COL6A6, and blank plasmid were purchased from Tolo Biotech (Shanghai, China).

### RT-qPCR

Total RNA was isolated from 0.2 g tissues or 5×10^5^ cells using Trizol reagent (QIAGEN, Germany). Tissues were ground in liquid nitrogen before the addition of extraction buffers, while cells were directly mixed with extraction buffers. And then, performed revere-transcribed reactions using PrimeScript^TM^ one-step real-time quantitative polymerase chain reaction (RT-qPCR) kit (TaKaRa, Japan). Real-time PCR was carried out using the Power SYBR Green PCR Master Mix (TaKaRa, Japan) and examined using the Bio-Rad System (Bio-Rad, USA). The sequences of the primers used for qPCR are presented in Table 2. The PCR reactions were carried out as follows: 95°C for 60 s, followed by 40 cycles of 95°C for 10 s and 57.5°C for 30 s. The 2^-ΔΔCt^ methods were applied to calculate the relative expression levels of COL6A6 and P4HA3. qPCR was performed in triplicate.

**Table 2:** Name and sequences of the primers

**Name**	**Primer sequences**
COL6A6	F: 5′-CCCAGGCCACAGATTTCCAT-3′
	R: 5′-TCCCACCCATCTGCCTGATA-3′
P4HA3	F: 5′-AAGTGGAGTACCGCATCAGC-3′
	R: 5′-TTGGTGACGTAGCATGGTCAA-3′
GAPDH	F: 5′-TCCAGTACGACTCCACCCAT-3′
	R: 5′-CGCCTTCTGCCTTAACCTCA-3′

F: Forward primer; R: Reverse primer

### CCK-8 assay

Cell Counting Kit-8 (Sigma, Japan) was used to assess the proliferation of both HP75 and AtT-20 cells. Both cells were seeded in 96-well plates at a density of 2.5×10^4^ cells per well and cultured with 5% CO_2_ at 37°C incubator for 2 h to allow cells to adhere. A total of 10 μL CCK-8 solution was added to each well and mixed, and cells were incubated for a further 2 h. A dual-wavelength microplate reader was used to measure proliferation at 450 nm (Beckman Coulter, USA). The assay was performed in triplicate.

### Flow cytometry analysis

Collected the samples mentioned above, washed with PBS, centrifuged at 800 *×g* for 6 min, suspended in ice-cold 70% ethanol/PBS, centrifuged at 800 *×g* for another 6 min, and suspended with PBS. Resuspended cells with 100 μL medium and added 5 μL of annexin V and 1 μL of propidium iodide according to the manufacturer’s instructions of Alexa Fluor 488 Annexin V/Dead Cell Apoptosis Kit (Thermo Fisher, USA), and incubated for 15 min at room temperature. BD LSR II flow cytometry was used to detect cells apoptosis (BD Biosciences, USA).

### Transwell invasion assay

Matrigel (BD Biosciences, USA) was coated the upper surface of polycarbonate filters. A total of 1×10^5^ cells/ml of cells were plated in 200 μL of the serum-free medium in the upper layer of the Transwell chamber (Corning, USA) and 800 μL medium supplemented with 10 % FBS was added to the bottom chamber. After 24 h of incubation, the cells which had invaded were fixed using 4% paraformaldehyde, rinsed three times with PBS, stained with 0.1% crystal violet for 10 min and rinsed three times with PBS. For quantification, 5 randomly selected fields were analyzed (magnification ×20).

### Wound healing assay

HP75 and AtT-20 cells (2×10^5^ cells/well) were treated with different reagents, seeded in six-well plates and cultured until they reached confluence. Wounds were made in the cell monolayer by making a scratch with a 20 μL pipette tip. Plates were washed once with fresh medium after 24 h in culture to remove non-adherent cells. Following this wash, plates were photographed under ×4 objective.

### Western blotting

Total protein was extracted for western blotting analysis. The PVDF membranes were incubated overnight at 4°C with primary antibodies against COL6A6 (1:1000, PA5-60958, Thermo, USA), P4HA3 (1:1000, ab101657, Abcam, Cambridge, UK), E-cadherin (1:1000, ab1416, Abcam, Cambridge, UK), N-cadherin (1:1000, ab202030, Abcam, Cambridge, UK), Vimentin (1:1000, ab193555, Abcam, Cambridge, UK), p-PI3K(p85)(1:1000, #ab191606, Abcam, Cambridge, UK), PI3K(p85) (1:1000, #ab191606, Abcam, Cambridge, UK), and p-Akt (), Akt (), and subsequently incubated with a horseradish peroxidase-conjugated secondary antibody (1:500, Abcam, Cambridge, UK). Signals were visualized using enhanced chemiluminescence reagent (Bio-Rad, USA).

### Nude mice model

Female BALB/c nude mice (4~5 weeks old) were purchased from the Kunming Institute of Zoology, Chinese Academy of Sciences and maintained in a pathogen-free facility. The model was approved by the Kunming Medical University. HP75 or AtT-20 cells (5×10^6^) transfected with NC or pcDNA COL6A6 were suspended in serum-free DMEM medium and then inoculated in left armpit of nude mice (10 nude mice in each group) at 5-6 weeks old to establish heterotopic transplanted tumor models of PA. In rescue experiments, the nude mice model was treated with PI3K-Akt activator IGF-1 (4 mg/kg) or equivalent amount of PBS by vein injection every two days for 2 weeks. Tumor growth was recorded every three days by measuring tumor length and width. After 4 weeks incubation, the mice were killed and the mice tumor tissues to be collected for further evaluated.

### Immunohistochemistry assay

The xenograft tissues were collected and fixed with formalin neutral solution of 10% volume fraction, paraffin-embedded and subsequently sectioned. A DAB horseradish peroxidase color development kit (Beyotime Institute of Biotechnology) alongside a Ki-67 antibody (1:2000, ab15580, Abcam, Cambridge, UK) were used to stain the samples at room temperature. Subsequently, slides were dyed with hematoxylin for 30 s, dehydrated and fixed, and then sealed with neutral glue. Stained images were observed and photographed under a fluorescence microscope (Olympus Corporation).

### Statistical analysis

Continuous variables are presented as the mean ± SD. The experimental data and image preprocessing were analyzed by SPSS 20 statistical software (IBM, USA) and GraphPad Prism7.0 software (La Jolla, USA), respectively. Besides, student’s t-test was used to analyze differences between the two groups, and the differences between multiple groups were compared by one-way ANOVA. Moreover, P<0.05 was identified as a statistically significant difference.
